# A Sub-Milliwatt Graphene-Based Thermal Conductivity Detector for On-Site Gas Analysis

**DOI:** 10.3390/s26113535

**Published:** 2026-06-03

**Authors:** Farhan Sadik Sium, Yunhao Peng, Steven Tran, Khandaker Reaz Mahmud, Md. Rabiul Hasan, Seungbeom Noh, Carlos H. Mastrangelo, Hanseup Kim

**Affiliations:** Department of Electrical and Computer Engineering, University of Utah, Salt Lake City, UT 84116, USA; farhan.sium@utah.edu (F.S.S.); yunhao.peng@utah.edu (Y.P.); steven.tran@utah.edu (S.T.); reaz.mahmud@utah.edu (K.R.M.); mdrabiul.hasan@utah.edu (M.R.H.); moses.noh@utah.edu (S.N.); carlos.mastrangelo@utah.edu (C.H.M.)

**Keywords:** graphene-based thermal conductivity detector, micro gas chromatography (µGC), low power sensing for on spot gas monitoring

## Abstract

**Highlights:**

**What are the main findings?**

- A multilayer graphene-based micro thermal conductivity detector (µTCD) is demonstrated as a distinct class of TCD, achieving sub-milliwatt operation (~151 µW) through an ultra-low thermal mass suspended graphene architecture, in contrast to conventional metal-based TCDs.
- The device generates chromatograms of VOCs (C5–C8) with ~350 ms transient response time and an estimated LOD of 190 ppm, while preserving chromatographic response comparable to conventional detectors.

**What are the implications of the main findings?**

- Establishes a fundamentally different operating regime for TCDs, where comparable sensing performance is achieved at orders-of-magnitude lower power than conventional metal-based designs.
- Opens a pathway for battery-powered, field-deployable micro gas chromatography systems with significantly reduced energy requirements.

**Abstract:**

This paper presents the design, fabrication, and characterization of a sub-milliwatt graphene-based micro thermal conductivity detector (µTCD) that utilizes a suspended multilayer graphene (MLG) bridge to sense volatile organic compounds (VOCs) in the gas phase based on their thermal transport properties. The graphene bridge is transferred onto a silicon chip with integrated microchannels using a photolithography-free process. By incorporating microchannel designs, this approach enables precise transfer of suspended MLG dimensions without conventional patterning steps. A key innovation of this work lies in the use of an ultra-low thermal mass suspended graphene architecture, which significantly increases temperature rise per unit input power, thereby enhancing sensitivity per unit power compared to conventional metal-based TCDs. The fabricated µTCD successfully produces chromatograms of multiple VOC species, closely matching those obtained using a standard flame ionization detector (FID). The device demonstrates an estimated limit of detection (LOD) of 190 ppm while consuming an average power of 151 µW under DC operation.

## 1. Introduction

On-site gas analysis plays a critical role in environmental monitoring and safety applications; however, achieving reliable in situ detection remains challenging due to strict requirements for low power consumption, compact design, and accurate gas identification for complex mixtures. These challenges are further compounded by limitations in sensor sensitivity, long-term stability, calibration drift, and susceptibility to environmental interferences such as temperature, humidity, and pressure variations [1]. Additionally, operation in harsh or remote environments introduces further engineering and material constraints, while the integration of on-site gas chromatography with automated systems demands robust and reliable sampling methods that are difficult to implement under such conditions [2]. Addressing these challenges is essential to advance on-site gas analysis technologies and enable more accurate, dependable, and scalable applications in pollution monitoring, industrial safety, and process optimization [1,2,3].

To address these challenges, the miniaturization of bulky gas monitoring systems has been enabled through the incorporation of microelectromechanical systems (MEMS) technology in the form of microfabricated pre-concentrators, micro-pumps, low-power ionization sources, and compact mass analyzers [4]. While gas chromatography (GC) is widely recognized for its ability to separate multiple chemicals, including volatile organic compounds (VOCs), integrating mass spectrometry (MS) into a portable system adds complexity due to the need for bulky components such as vacuum components, ionization sources, and high-precision detectors [4,5]. Despite ongoing challenges such as power efficiency, vacuum system miniaturization, and robust data processing, miniaturized GC systems remain increasingly viable for on-site gas analysis in diverse settings [5,6].

Within these miniaturized systems, a critical component of a micro gas chromatography platform is the gas detector. As shown in Table 1, various miniaturized gas detectors, such as Thermal Conductivity Detectors (TCDs), Chemiresistors, Flame Ionization Detectors (FIDs), and Photoionization Detectors (PIDs) have been developed to enhance detection capabilities. However, traditional systems face several limitations. Chemi resistors exhibits signal degradation over time (10–20% per cycle) [7,8,9], while PIDs are constrained by high power consumption (1–10 W), bulky hardware requirements (≥100 cm^3^) [10,11,12], and the need for a high-intensity ultraviolet (UV) ionization lamp that demand substantial energy and optical isolation, making them unsuitable for compact, battery-powered, on-site applications. Recent efforts have sought to improve PID selectivity through material integration, such as Zeolite-assisted adsorption layers for targeted VOC detection [13]. However, these approaches remain dependent on ionization-based transduction and associated high-power hardware [14]. Similarly, FIDs require in situ flame generation, which restricts their use in environments containing combustible gases [15,16].

**Figure 1:**
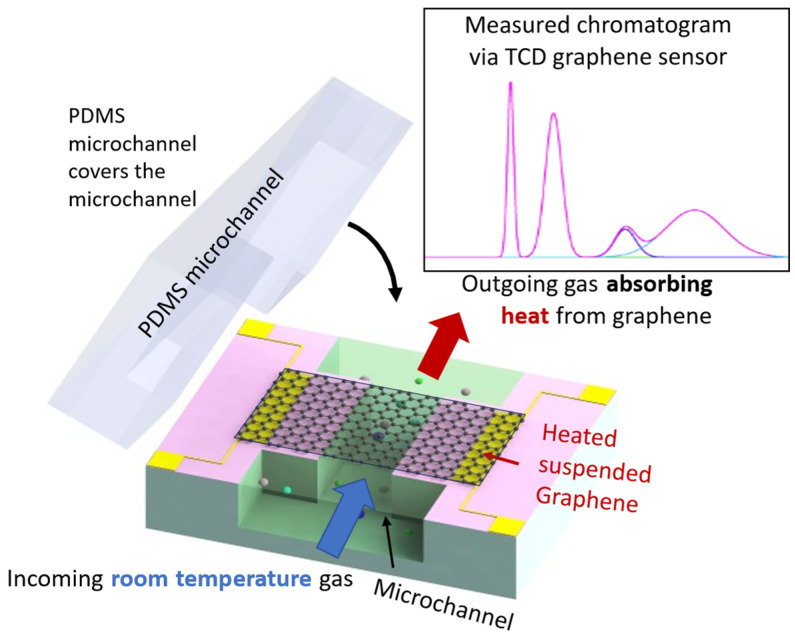
3D illustration of a graphene-based µTCD utilizing freestanding multiple-layer graphene (MLG) as a sensing resistor. The MLG layer provides high strength to remain freestanding without other supporting layers. (Blue indicates the relatively cooler incoming room-temperature gas, whereas red indicates the outgoing gas after slight heating due to heat transfer from the suspended graphene heater).

**Figure 2:**
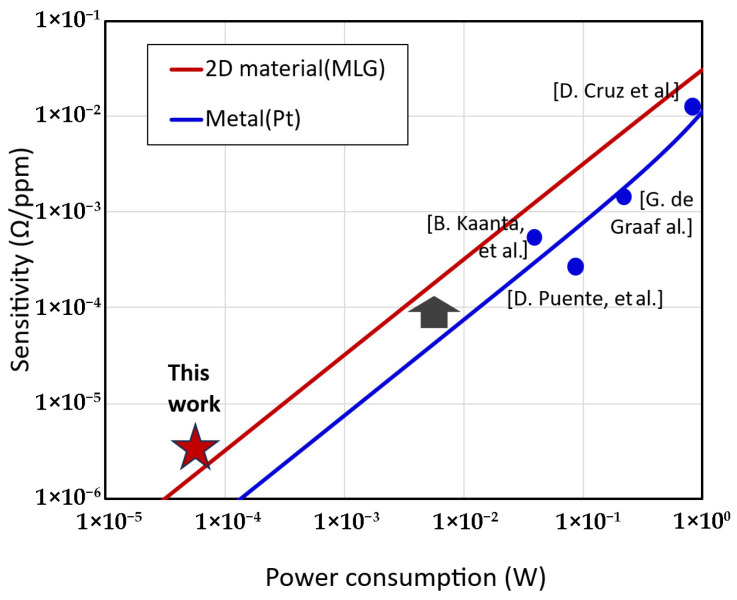
Sensitivity-power trade-off for graphene-based µTCDs compared to conventional metal-based TCDs [17,18,27,28]. Lumped modeling [20] (blue lines) and experimental data from the literature (symbols) show that graphene µTCDs (red star) operate at significantly lower power levels (~10^−4^ W), reflecting a tradeoff between reduced power consumption and lower sensitivity.

Among available detector technologies, thermal conductivity detectors have been extensively investigated due to their potential for miniaturization (<10 cm^3^), tunable power consumption (0.1 mW–1 W), and intrinsic safety, as their operation does not rely on plasmas or electrical discharges. Most of these TCDs are based on metallic (e.g., Pt, Ni, Cr) thermistors or heaters [17,18,19,20]. However, the main limitation of TCDs lies in their dependence on power to achieve higher sensitivity and lower limits of detection (LOD). Mahdavifar et al. demonstrated that the sensitivity of TCDs increases with power, following a trend where higher power yields better sensitivity [21]. The device structure and measurement concept of the graphene-based µTCD developed in this work are shown in Figure 1. The sensitivity–power comparison of reported TCDs is summarized in Figure 2.

Despite these advantages, conventional metal-based µTCD designs often suffer from high power consumption, prompting the exploration of graphene as an alternative material. Graphene’s atomically thin structure (~0.34 nm for a monolayer), combined with its exceptional mechanical strength (Young’s modulus > 1 TPa) and high thermal conductivity (~2000–5000 W/m·K) [22,23], enables the formation of suspended sensing elements with extremely low thermal mass. For instance, replacing a 50 nm platinum membrane supported by a 500 nm Si_3_N_4_ layer with a monolayer graphene membrane in a freestanding µTCD (100 × 100 µm^2^ footprint) reduces thermal mass by three orders of magnitude, from 3.52 × 10^−9^ to 5.32 × 10^−12^ J·K^−1^ [23]. This reduction directly translates to improved power efficiency, making graphene-based µTCDs highly suitable for battery-operated µGC systems and portable on-site gas analysis. Additionally, it must be noted that graphene’s high surface-to-volume ratio enhances gas–molecule interactions, resulting in stronger signal responses and improved detection performance [21].

Going further in-depth with this approach, this paper presents the development of a graphene-based µTCD, detailing its fabrication, operation, and validation while leveraging the temperature resistance correlation of graphene to achieve low power consumption and effective VOC detection, allowing for a portable, low-power, on-site gas analysis system. Note that the traditionally challenging task of patterning a freestanding graphene layer was successfully addressed using a novel geometry-induced pattering technique, eliminating the need for conventional photolithography and enhancing scalability for practical applications.

## 2. Operation and Modeling

### 2.1. Operation Principle

Figure 1 presents the structural design and operating principle of the graphene-based µTCD, highlighting a multilayer graphene (MLG) bridge suspended across the pinched region of a fully enclosed microchannel without the need for supporting layers. The MLG layer is strategically positioned near the mid-height of the microchannel to enhance interaction with flowing gas molecules while preserving thermal isolation from the substrate, thereby enabling power-efficient operation. The suspended graphene bridge is interfaced with four electrodes, where one pair supplies an electrical bias to induce Joule heating and the other pair monitors the resulting resistance changes. Upon current application, Joule heating increases the temperature of the graphene above that of the surrounding gas, and the generated heat is dissipated predominantly through conduction into the gas within the microchannel.

As gas molecules flow through the channel, there is a heat exchange with the graphene bridge. The rate of heat dissipation depends on the thermal conductivity of the gas, resulting in a gas-dependent temperature of the graphene. This temperature variation is transduced into an electrical signal through the temperature dependence of graphene resistance (Equation (1)):

(1) $$R=R_{0}\left(1+\alpha (T-T_{\infty })\right),$$

where $R_{0}$ is the baseline resistance at ambient temperature $T_{\infty }$, and α is the temperature coefficient of resistance.

Due to the suspended configuration and nanoscale thickness of graphene, the thermal mass of the sensing element is significantly reduced compared to conventional metal-based microbridges. This enables larger temperature changes at low input power and allows the device to operate effectively in the sub-milliwatt regime.

### 2.2. Lumped Thermal Model

To describe the thermal response of the suspended graphene bridge, a lumped electrothermal model was employed based on prior micro-TCD studies [21]. This approach assumes that the temperature across the sensing element is spatially uniform (Supplementary Figure S1), which is justified by the high in-plane thermal conductivity [22,23,24] of graphene relatives makes near-uniform temperature distribution. The transient thermal behavior is governed by the energy balance between heating and dissipation, is shown in Equation (2):

(2) $$P_{0}=C_{th}\frac{dT}{dt}+K_{tot}(T-T_{\infty }),$$

where $P_{0}=I^{2}R_{0}$ is the applied electrical power, $C_{th}=\rho VC_{p}$ is the thermal capacitance of the graphene bridge, and $K_{tot}$ is the total thermal conductance. Here, ρ is the density, V is the volume, and $C_{p}$ is the specific heat capacity of graphene.

At microscale dimensions, heat transfer is dominated by gas-phase conduction, while convection is suppressed due to scaling effects. Natural convection is negligible because the Rayleigh number scales as $Ra\propto L^{3}$, and for lengths on the order of $10^{-4}–10^{-3}$ m, $Ra\ll 10^{3}$, which is below the threshold required for buoyancy-driven flow [25]. So thermal transport occurs primarily through conduction rather than bulk fluid motion.

Forced convection is also minimal under the present operating conditions. At low flow rates (~0.5 sccm), the Peclet number remains small (Pe≪1), indicating that advective heat transport is much weaker than conductive transport. Therefore, both natural and forced convection contribute negligibly, and heat transfer in the µTCD is governed primarily by gas-phase conduction [21,26]. The gas-dependent thermal conductance is expressed as shown in Equation (3):

(3) $$K_{gas}=k_{gas}\frac{A_{conv}}{L_{gas}},$$

where $k_{gas}$ is the thermal conductivity of the gas. Solving the transient equation yields an exponential temperature response, as shown in Equation (4):

(4) $$T(t)-T_{\infty }=\frac{P_{0}}{D}\left(1-e^{-t/\tau }\right),$$

where

(5) $$D=K_{tot}-\alpha P_{0};\tau =\frac{C_{th}}{D},$$

Note that the corresponding resistance change is given by Equation (6) and that the sensitivity of the device to gas thermal conductivity is obtained by differentiating the resistance response and found as Equation (7):

(6) $$\Delta R(t)=\alpha R_{0}\frac{P_{0}}{D}\left(1-e^{-t/\tau }\right),$$

(7) $$\frac{d(\Delta R)}{dk_{gas}}=\alpha R_{0}P_{0}\left(\frac{A_{conv}}{L_{gas}}\right)\left[-\frac{1-e^{-t/\tau }}{D^{2}}+\frac{t\;e^{-t/\tau }}{D\;C_{th}}\right]$$

This expression indicates that the sensor response is governed by both the thermal conductance and the thermal capacitance of the system.

### 2.3. Modeling Results

Figure 2 compares the sensitivity–power relationship of reported µTCDs and highlights the distinct scaling behavior between conventional metal-based devices and graphene-based implementations. Traditional metal TCDs exhibit a near-linear trend in log scale (blue line), where sensitivity increases with input power as greater Joule heating produces larger temperature differentials, resulting in most devices operating within the milliwatt to watt range to achieve measurable performance. In contrast, graphene-based devices (red line) shift this scaling toward significantly lower power operation due to their reduced thermal capacitance and enhanced thermal isolation. The device demonstrated in this work (star marker) operates at 10^−4^ W with a corresponding lower sensitivity of 3.1 × 10^−5^ Ω/ppm. This position is in a regime where conventional metal TCDs are largely ineffective due to their high thermal mass. As a result, the shift in sensitivity–power trade-off, enables µTCD operation in the ultra-low-power domain.

While the scaling trends provide a useful comparative framework, the underlying thermal model is based on a lumped approximation [21] that captures the dominant conduction-driven behavior of the device. It does not explicitly account for detailed microchannel transport effects such as flow profile, thermal entrance development, or three-dimensional heat spreading, which are instead incorporated into an effective thermal conductance term.

## 3. Materials and Fabrication Process

### 3.1. Free Standing Graphene Transfer

The core component of the TCD-based µGC is the suspended multilayered graphene. To integrate this graphene into the gas-sensing chip, a multi-step method was employed for transferring and patterning. This process enables the precise placement of a relatively large (100 × 100 µm^2^) freestanding multilayer graphene membrane over a microscale trench using multilayer graphene grown on copper foil purchased from ACS Material, LLC (Pasadena, CA, USA). According to Equations (8) and (9), the stiction force (F_D_ and F_a_) between the graphene and substrate increases as the overlapped area increases while the microchannel in the substrate is constant.

(8) $$F_{D}=\frac{1}{2}\rho u^{2}C_{D}A_{D}$$

(9) $$F_{a}=2\alpha A_{a}$$

In Equations (8) and (9), ρ is the fluid density, u is the fluid velocity, $C_{D}$ is the drag coefficient, and $A_{D}$ is the projected area exposed to flow. In Equation (9), α represents the interfacial adhesion energy, and $A_{a}$ is the contact area between graphene and the substrate. Both forces scale linearly with area, indicating that increased overlap leads to higher interaction forces, promoting stiction in supported regions while enabling suspension over the trench.

#### 3.1.1. Graphene Transfer Process

**Figure 3:**
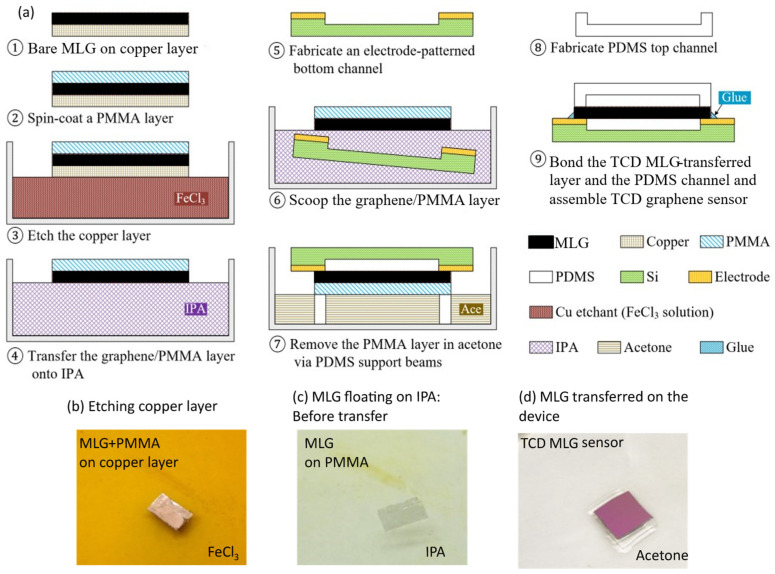
(**a**) Schematic illustration of the fabrication process for the suspended multilayer graphene (MLG) µTCD sensor. The process includes PMMA-assisted transfer of MLG from a copper substrate via FeCl_3_ etching, transfer through IPA, placement onto a prefabricated electrode-patterned microchannel, removal of the PMMA support layer, and final assembly with a PDMS top channel. (**b**) Optical image showing the MLG/PMMA stack during copper etching in FeCl_3_ solution. (**c**) Optical image of the MLG/PMMA film floating on IPA prior to transfer. (**d**) Final assembled device showing the MLG integrated onto the microchannel to form the TCD sensor.

The graphene transfer process is illustrated in Figure 3, where the process begins with multilayer graphene (MLG) grown on a copper substrate (Step 1), followed by spin-coating a PMMA A4 resist layer (MicroChem Corp., Newton, MA, USA) to provide mechanical support during transfer (Step 2).Subsequently, the PMMA layer was spin-coated at 2000 rpm for 45 s and soft baked at 80 °C for 2 min. The relatively low baking temperature (below the PMMA glass transition temperature of ~110 °C) was used to minimize thermal stress and prevent damage to the MLG layer. Note that at relatively low temperature, the residues in the processes [29] are minimized, thus mitigating potential defects. Also note that the MLG used in this work was commercially sourced as CVD-grown graphene on copper foil with a PMMA-assisted transfer process (ACS Material). According to manufacturer specifications, the material corresponds to multilayer graphene with a thickness typically in the range of ~1–10 nm (corresponding to multiple stacked graphene layers), representing multiple stacked graphene layers, and exhibits a sheet resistance on the order of <600 Ω/sq prior to transfer [30].

After the baking process, the PMMA/MLG/copper stack was placed on a ferric chloride (FeCl_3_) solution with the copper side facing downward to etch away the copper substrate (Step 3). Approximately 30 min afterwards, the copper layer was fully removed, leaving the PMMA/MLG film floating on the solution. The solution was subsequently replaced with deionized water and then isopropyl alcohol (IPA) (Step 4). The use of IPA, with lower surface tension (~0.024 N·m^−1^ compared to ~0.072 N·m^−1^ for water), reduced capillary forces and minimized the risk of graphene film rupture.

In parallel, a device chip with a prefabricated electrode-patterned bottom microchannel was prepared (Step 5). The floating PMMA/MLG film was then carefully scooped and transferred onto the device chip, aligning the graphene layer over the microchannel region (Step 6). The sample was then left to dry under ambient conditions for approximately two hours to promote adhesion between the graphene layer and the substrate. Following the transfer, the PMMA support layer was removed by immersing the sample in acetone (Step 7), leaving the graphene layer intact and partially suspended over the microchannel trenches. Finally, a PDMS top channel was fabricated (Step 8) and bonded onto the graphene-transferred device using adhesive bonding (Step 9), completing the assembly of the graphene-based µTCD sensor.

#### 3.1.2. Evaluation of the Transferred Graphene Film

The transfer yield was monitored to determine the maximum transferable size of the suspended MLG films. To assess this, the transfer process was repeatedly performed over square trenches of varying sizes, ranging from 30 to 150 µm, on custom fabricated silicon trench chips. For each trench size, 100 square trenches were patterned and etched onto the silicon die, enabling a comprehensive evaluation of the transfer success rate.

**Figure 4:**
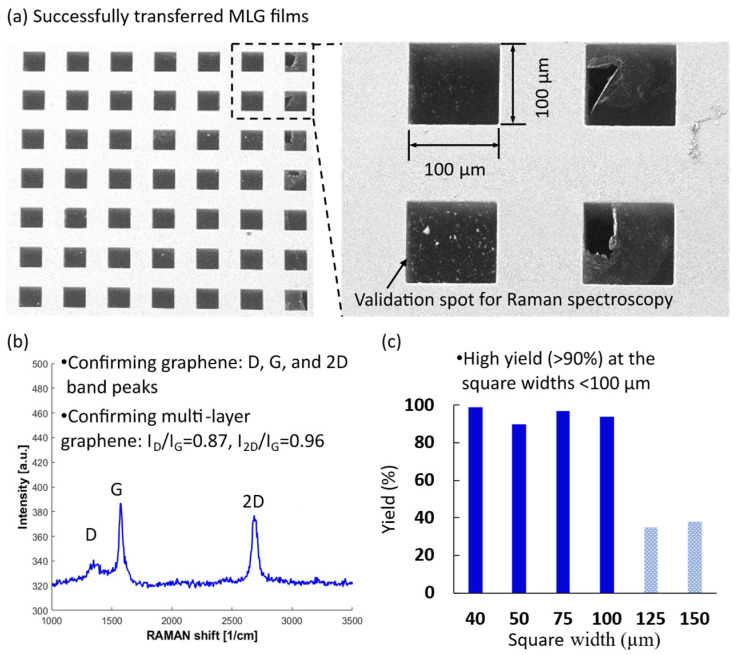
Transfer the result of MLG onto a Si/SiO_2_ chip with square-shaped trenches. (**a**) SEM image of freestanding MLG covering 100 × 100 µm^2^ square-shaped trenches. There was a total of 100 trenches, where 94 of them have complete MLG coverage with no visible pinholes or cracks; to the right is the closeup SEM image of 4 MLG films, showing both intact suspended films and partially ruptured films; (**b**) Raman spectrum (at 499 nm wavelength, 0.5 s integration) of a randomly chosen spot on the ruptured MLG, showing graphene’s signature peaks, the D, G, and 2D; the D peak indicates defects from MLG. (**c**) the yield for the number of trenches having intact suspended MLG film vs. various trench sizes.

The measurement results indicated that the MLG film transfer achieved a high yield of ≥90% for square trenches ranging from 40 × 40 µm^2^ to 100 × 100 µm^2^. However, the yield significantly dropped to ≤40% for trenches exceeding 125 × 125 µm^2^ footprints (Figure 4c). This yield decrease was expected as the size of the freestanding MLG increased as larger surface areas were subjected to greater surface tension during liquid drying. Thus, the sudden drop in yield at 125 µm establishes the current limitation of this study for stable and scalable manufacturing.

As shown in Figure 4b, the Raman spectrum exhibits the characteristic D (~1350 cm^−1^), G (~1580 cm^−1^), and 2D (~2700 cm^−1^) peaks commonly reported for graphene-based materials [31]. A D peak is commonly observed in CVD-transferred graphene due to defect-activated scattering from growth- and transfer-induced disorder. The Raman spectrum confirms the presence of graphene after transferring through the characteristic G and 2D peaks and their expected spectral positions, while the sensing mechanism is governed by electrothermal response [32].

### 3.2. Device Fabrication

**Figure 5:**
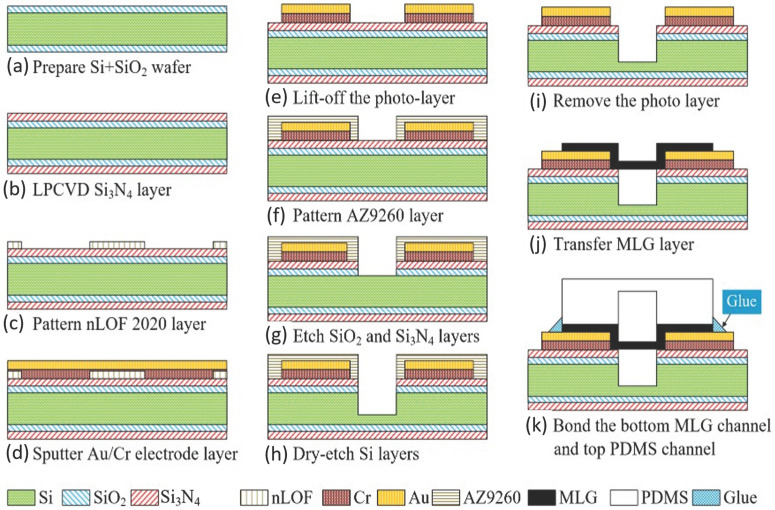
Schematic illustration of the microfabrication process for the µTCD device: (**a**) preparation of Si/SiO_2_ wafer; (**b**) LPCVD of Si_3_N_4_ layer; (**c**) photolithographic patterning using nLOF 2020 resist; (**d**) sputter deposition of Au/Cr electrode layer; (**e**) lift off to define electrodes; (**f**) patterning of AZ9260 photoresist to define the microchannel region; (**g**) etching of SiO_2_ and Si_3_N_4_ layers; (**h**) deep etching of the Si substrate to form the suspended channel; (**i**) removal of the photoresist; (**j**) transfer of multilayer graphene (MLG) onto the device; and (**k**) bonding of the bottom MLG channel with a PDMS top channel to complete the µTCD sensor.

Figure 5 illustrates the microfabrication process flow for constructing a suspended graphene-based µTCD. The process began with an n-type, 525 µm thick, 4-inch silicon wafer purchased from University Wafer, Inc. (South Boston, MA, USA), featuring a 250 nm thick thermal oxide layer on both sides (step 1). First, a 250 nm thick low-stress Si_3_N_4_ thin film was deposited on the wafer surface using low-pressure chemical vapor deposition (LPCVD, Expertech, Scotts Valley, CA, USA) to provide passivation on the wafer’s backside (step 2). The wafer was then coated with hexamethyldisilane (HMDS; Sigma-Aldrich, St. Louis, MO, USA) and negative AZ nLOF 2020 (Merck KGaA, Darmstadt, Germany) photoresist (2.6 µm) before being patterned via standard photolithography (step 3). Next, a blanket sputtering process was used to deposit Cr/Au (50/150 nm) films on the wafer surface. The areas with patterned photoresist underneath were subsequently removed using a lift-off process in an ultrasonic acetone bath, defining the electrodes (steps 4 and 5). To create the microchannel patterns, a second HMDS/AZ9260 (Merck KGaA, Darmstadt, Germany) (7.6 µm) coating was applied and developed (step 6). The frontside Si_3_N_4_ and SiO_2_ layers were then etched using reactive ion etching (RIE) and buffered oxide etching (BOE; Transene Company, Inc., Danvers, MA, USA), respectively (step 7). The exposed bulk silicon was etched using deep reactive ion etching (DRIE; Oxford Instruments Plasma Technology Ltd., Bristol, UK), forming 150 µm deep microchannels (step 8). After the photoresist was removed (step 9), the MLG bridge was then transferred onto the microchannels using a freestanding MLG membrane, though it randomly ruptured during the transfer process.

### 3.3. Polydimethylsiloxane (PDMS) Capping

The transferred membrane was enclosed by placing a Polydimethylsiloxane (PDMS) substrate over it, which had been previously fabricated using a patterned SU-8 photoresist mold. This PDMS cap was manually aligned to the chip under a microscope and gently pressed down to ensure proper bonding. Finally, silicone glue was applied around the edges to achieve hermetic sealing.

PDMS was selected as the cover material due to its chemical inertness and excellent thermal stability. Recent research has demonstrated that adsorption in PDMS could be negligible when only minimal amounts of analyte are present and insufficient time is allowed for diffusion [33]. In our study, we used a small VOC pulse (maximum 0.1 µL) to generate the desired chromatogram, with the longest pulse width lasting less than five seconds. These conditions justify the PDMS cap’s inability to absorb rapidly flowing, low concentrations of VOCs, ensuring minimal interference with the detection process.

### 3.4. Geometry-Defined Graphene Patterning

Patterning a freestanding graphene membrane has been challenging using conventional liquid-based methods, such as lithography-based patterning or sacrificial layer etching due to the drying process. During drying, the liquid surface tension often exerts forces that lead to unintended rupture, making precise patterning difficult. In this study, an approach was followed by leveraging abrupt dimensional changes in trenches underneath the membrane, achieving controlled rupture for patterning. This approach is consistent with prior studies that have reported capillary-induced deformation and failure of suspended membranes during drying [34]. During solvent evaporation, the suspended MLG membrane is subjected to capillary pressure generated by the liquid meniscus, which can be expressed as Equation (10) [34]:

(10) $$P_{cap}=\frac{4\gamma sin\theta }{D}$$

where γ is the surface tension, θ is the contact angle, and D is the characteristic liquid-filled dimension.

This pressure acts as a distributed load on the membrane. Specifically, as the MLG layer was transferred in liquid and subsequently dried, it experienced mechanical rupture over the large-width trench regions due to surface tension pulling on the freestanding membrane. However, this effect did not occur in narrow-width trench regions, where the structural integrity of the MLG membrane was preserved. The resistance to rupture at smaller trench widths can be explained by the dependence of stress on the suspended span. Under capillary loading, the induced stress scales as Equation (11) [34]:

(11) $$\sigma \propto \frac{P_{cap}a^{2}}{t^{2}}$$

where a is the trench width and t is the membrane thickness. As a result, narrower trenches generate significantly lower stress and deformation, allowing the membrane to remain intact. This technique successfully maintains a freestanding MLG membrane in narrow or pinched regions < 40 µm in width, while consistently rupturing membranes in wider regions exceeding 150 µm, in a repeatable and controlled manner. The results align with the transfer characterization findings shown in Figure 3, demonstrating the approach for precision graphene patterning.

**Figure 6:**
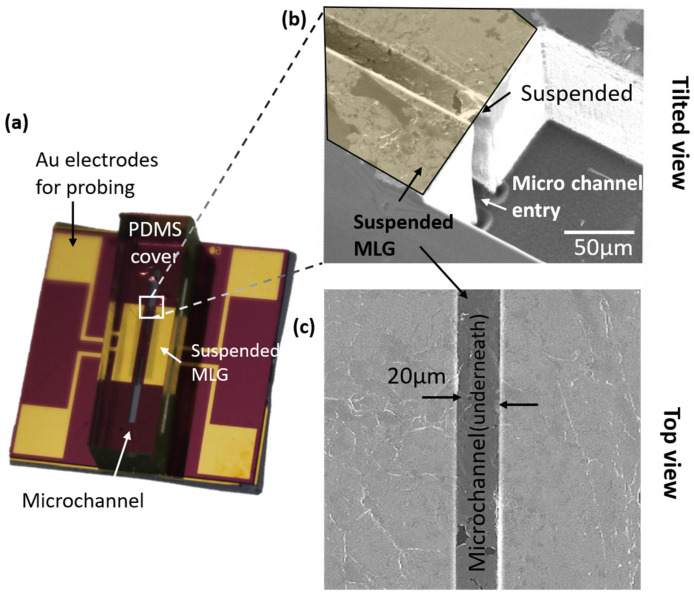
Fabricated graphene µTCD device. Optical image (**a**) shows the PDMS-covered structure with a suspended MLG sensing element. SEM images (**b**) reveal the suspended graphene over the pinched microchannel and the ~20 µm-wide channel.

**Figure 7:**
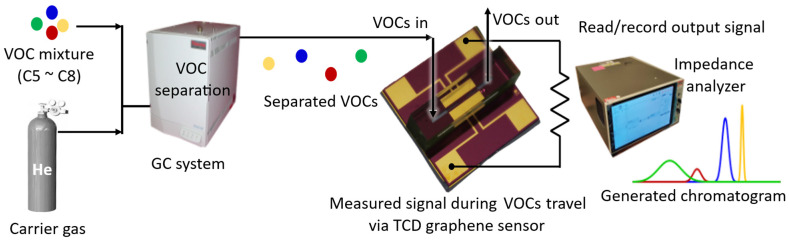
Experimental setup for VOC detections. VOCs (e.g., C5, C6, C7, C8) were injected through a commercial benchtop GC system (Thermal Scientific Focus GC) to graphene-based µTCDs utilizing suspended MLG as the sensing resistor. A parameter analyzer (Keithley 4200A-SCS, Keithley Instruments, Cleveland, OH, USA) measured the device resistivity variation at 10 Hz while applying 500 mV DC.

To define the freestanding MLG membrane, a pinched microchannel was microfabricated to exploit stress localization at the interface between suspended and anchored regions under capillary loading during the liquid drying process [35]. In the anchored regions, the graphene is supported by the substrate, imposing no deflection (w≈0). Thus, stress is very low in the anchored region. In contrast, the suspended regions deform under the same capillary pressure, resulting in higher stress that increases with span. At the transition between anchored and suspended regions, this abrupt change in mechanical constraint produces a strong spatial variation in deformation, leading to localized stress concentration. Consequently, rupture is preferentially initiated near this interface and propagates into the wider suspended region, thereby defining the pattern boundary in a deterministic manner. The fabricated device is shown in Figure 6. This approach successfully defined freestanding MLG membranes using pinched microchannels of varying dimensions, including 20 × 825 µm^2^ (Figure 7), 30 × 550 µm^2^, and 40 × 410 µm^2^. Note that all structures maintained a consistent footprint of approximately 1.65 × 104 µm^2^.

Furthermore, by fixing the width at 20 µm while varying the length from 200 µm to 1200 µm, the resistance of the suspended MLG exhibited a decreasing trend, ranging from 1.8 kΩ to 0.58 kΩ. This behavior aligns with the expected relationship in an ideal resistor, where resistance is inversely proportional to the cross-sectional area observed from the electrodes on both sides. These results demonstrate the feasibility of modulating graphene resistance through geometric design, offering a scalable and tunable approach for optimizing MLG-based microdevices. The final device is shown in Figure 6, with PDMS cap and suspended MLG on top of microchannel.

## 4. Testing Methodology

### 4.1. Temperature and Flow Calibration

Since the output of the TCD is influenced by temperature variance, it is crucial to characterize the temperature effect, either from the direct heating of MLG or from the cooling of MLG via carrier gas flow, to establish the baseline for calibration. The output resistance of the manufactured graphene TCD was observed. At the same time, the TCD was exposed to N_2_ gas flow from 10 sccm up to 30 sccm at 5 sccm intervals, and temperature ranges from room temperature to 80 °C with 10 °C intervals, to characterize the device response to both different temperature changes and gas flow rate. During the temperature exposure, the µTCD was located within a testing chamber (MPS-PT; Nextron Co., Ltd., Busan, Republic of Korea) that controlled the temperature with an integrated Peltier heating stage. The heating stage ramped up the temperature at a rate of 10 °C·min^−1^ and then held the temperature at each target point for 5 min until the next interval. The chamber contained two probes to monitor transient electrical resistance, and the probes were connected via feedthroughs to a parameter analyzer (Keithley Instruments, LLC, Solon, OH, USA).

To evaluate the repeatability of the temperature calibration, the device was subjected to periodic temperature cycling using a Nextron Peltier-controlled stage. The temperature was alternated between 25 °C and 35 °C, with each temperature held for a duration sufficient for the device to reach steady-state conditions. This heating-cooling sequence was repeated for at least three cycles to examine recovery behavior and cycle-to-cycle reproducibility of the resistance response.

During the flow testing gas, the chamber with the µTCD was fixed to room temperature and supplied with N_2_ at a precisely controlled flow rate using a GF040 thermal mass flow controller (Brooks Instrument, Hatfield, PA, USA).

### 4.2. Repetable Detection of VOC

To evaluate VOC detection in a repeatable manner, the sensor was placed inside a Nextron gas testing chamber. A constant bias voltage was applied using the force-SMU of a Keithley 4200S parameter analyzer. After applying the bias, the sensor output was allowed to stabilize until a steady-state baseline was reached under pure He flow.

Once stabilization of the baseline resistance was achieved, gas pulses of 5 s duration were introduced using programmable mass flow controllers. A premixed pentane (0.5% *v*/*v*) stored in a Tedlar bag was diluted with He using two independently controlled mass flow controllers operated at equal flow rates of 2.5 sccm, resulting in a total flow rate of 5 sccm and a concentration of ~2500 ppm. The resistance was measured in Kiethley 4200S (sampling frequency 6 Hz). Details of the test setup and the mass flow-based concentration calculation are provided in Supplementary Figure S2.

### 4.3. VOC Deployment and Chromatogram Generation

To validate the feasibility of detecting VOCs at low power consumption, the fabricated µTCD was exposed to a mixture of liquid pentane (C5), hexane (C6), heptane (C7) and octane (C8), while the input power was maintained at 500 mV, and its output signal was monitored. As shown in Figure 7, the input gas flow of the µTCD was controlled by a commercial benchtop GC system (Scientific Thermal Focus GC), while the parameter analyzer electrically monitored the output signal of the µTCD. The benchtop GC injected the mixture of liquid C5, C6, C7 and C8 in the 1:1:1:1 ratio on top of a carrier gas, helium, at 0.5 sccm with a gas split ratio of 1:70 and at 40 °C constant oven temperature. The injected mixture went through a 50 cm long column coated with OV-1 stationary phase for separation before it reached the µTCD. Meanwhile, the parameter analyzer measured the µTCD resistance outputs at a sampling rate of 10 Hz. For comparison purposes, the identical testing was repeated with a commercial flame ionization detector (FID).

### 4.4. Sensitivity Characterization

To characterize the device sensitivity to a target VOC at a given power consumption, the µTCD was exposed to pentane (C5) with injection volumes ranging from 0.01 µL to 0.1 µL. The analyte was introduced using a commercial GC system operating in split injection mode (1:70), where the injected liquid is rapidly vaporized in the heated injector and transported by the helium carrier gas prior to column separation.

The corresponding gas-phase concentrations were estimated using a first-order mass balance approach [36]. Specifically, the injected liquid volume was first converted to moles using VOC density and molecular weight as $n=\frac{\rho V_{liq}}{M}$, followed by dilution through the split ratio as $n_{col}\approx \frac{n}{1+S}$ (s = split ratio) and subsequently normalized by the carrier gas molar flow to obtain the concentration given by $C_{ppm}=\frac{n_{col}}{{\dot{n}}_{gas}\;t}\times 10^{6}$, where ${\dot{n}}_{gas}$ is the carrier gas molar flow rate and t represents the effective temporal width (2.5 s) of the analyte plug. Based on this approach, the injections correspond to estimated gas-phase concentrations in the range of 475 ppm to 4759 ppm.

The raw µTCD output signal was post-processed to extract key sensing parameters from each injection event. First, the baseline was corrected using a running average window of 30 data points, corresponding to 3 s at a sampling rate of 10 Hz. The processed signal was then fitted using Gaussian functions to determine peak positions and amplitudes. From these fitted signals, ΔR was defined as the difference between the peak resistance and the baseline resistance prior to injection. This parameter represents the magnitude of the thermal response induced by the analyte and serves as the primary sensing signal for quantifying gas concentration.

In addition to the peak amplitude, the dynamic behavior of the device was evaluated through its transient response. The transient response time was defined as the time required for the resistance signal to reach 63.2% of the total resistance change, corresponding to the first-order thermal time constant. This metric is critical for chromatographic applications, as it determines the ability of the detector to resolve closely spaced peaks.

The extracted ΔR values were then correlated with the corresponding analyte concentration to determine device sensitivity. To evaluate signal reliability, the signal-to-noise ratio (SNR) was calculated using the root-mean-square (RMS) noise of the baseline signal. The limit of detection (LOD) was subsequently determined based on a predefined SNR threshold, representing the minimum analyte concentration that can be reliably distinguished from noise.

### 4.5. Power Calculations During Chromatography

To determine the electrical power consumption during chromatographic measurements, the resistance of the device was measured in situ while the µTCD was connected to a DC bias source (Keithley 4200A-SCS parameter analyzer). A constant DC voltage bias was applied across the suspended MLG bridge, and the resulting current was simultaneously measured by the instrument.

The device resistance was then extracted using Ohm’s law, R=V/I, based on the applied voltage and measured current.

## 5. Experimental Results and Discussion

### 5.1. Baseline Calibration

**Figure 8:**
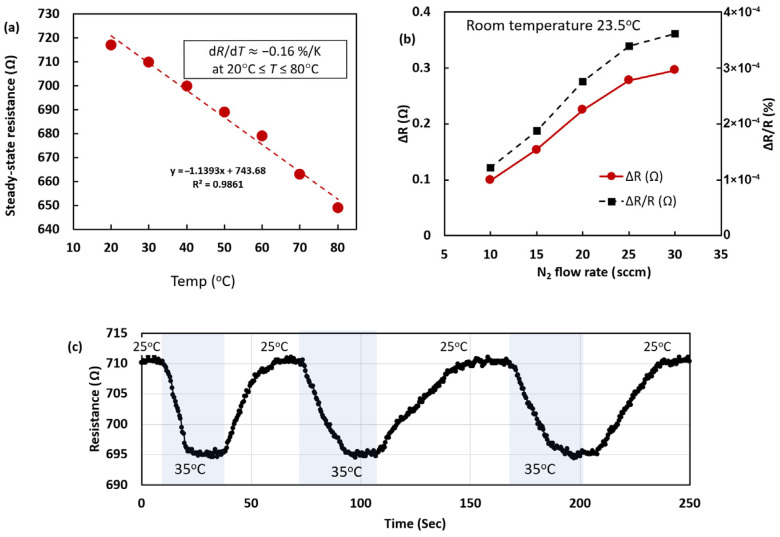
Calibration of the graphene-based µTCD. (**a**) Temperature calibration showing the resistance variation with temperature from 20 °C to 80 °C, exhibiting a linear response with a temperature coefficient of resistance (TCR) of −0.16%/K. (**b**) Flow calibration showing the ΔR and normalized response (ΔR/R) as a function of N_2_ flow rate (10–30 sccm), demonstrating an increase in signal with increasing flow. (**c**) Three cycles of temperature variation from 25 °C to 35 °C showing repetitive recoverable response.

The calibration baselines of the µTCD output were established for both temperature and flow rates. The fabricated µTCD with a 30 × 550 µm^2^ suspended MLG footprint was characterized over a temperature range of 20–80 °C in 10 °C intervals, with the device held at each setpoint for 5 min to ensure thermal equilibration before recording the resistance. As shown in Figure 8a, the steady-state resistance decreased from 717 Ω at 20 °C to 648 Ω at 80 °C, exhibiting a linear dependence on temperature. A least-squares fit through the equilibrium points yielded a normalized temperature coefficient of resistance (TCR) of α ≈ −0.16%/K (dR/dT = −1.14 Ω/K, R^2^ = 0.986) over the 20–80 °C range.

To further evaluate recovery and repeatability, the device was subjected to repeated temperature cycling, as shown in Figure 8c. The resistance consistently returns to its initial baseline after each cycle, confirming reversible behavior and complete recovery under cyclic calibration conditions. Similar behavior has been reported in graphene-based temperature sensors, where resistance returns to baseline following thermal perturbation, demonstrating repeatable and reusable operation without degradation [37].

The baseline response with respect to gas flow rate was evaluated under N_2_ flow, as shown in Figure 8b. As the flow rate increased from 10 to 30 sccm, both the absolute resistance change (ΔR) and normalized response (ΔR/R) increased monotonically. Specifically, ΔR increased from 0.1 Ω to 0.3 Ω, while ΔR/R exhibited a corresponding increase, indicating enhanced heat dissipation from the graphene bridge.

This behavior is attributed to increased convective heat transfer at higher flow rates, which enhances the thermal conductance between the graphene bridge and the surrounding gas. As a result, the bridge temperature decreases, leading to a measurable resistance change due to the negative TCR of graphene. The monotonic and repeatable response confirms that the device is sensitive to flow-induced thermal variations and establishes a stable baseline for subsequent gas sensing measurements.

### 5.2. Repitative Detection of the Same VOC Undersimilar Condtion

**Figure 9:**
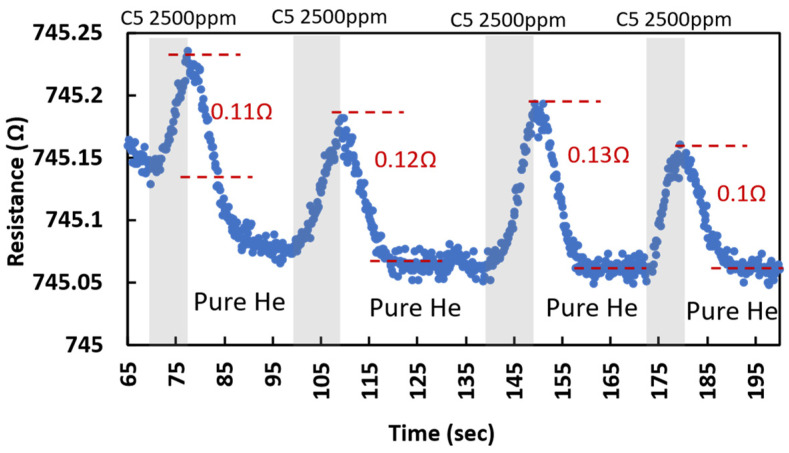
Repetitive response of the MLG TCD under similar (C5: 2500 ppm) gas concentration.

As shown in Figure 9, the sensor exhibited a consistent response under repeated exposure to identical VOC conditions. Four consecutive gas exposure cycles were performed using pentane (C5), and the corresponding ΔR were measured as 0.11 Ω, 0.12 Ω, 0.13 Ω, and 0.1 Ω, respectively. These values yielded an average response of 0.11 Ω with a standard deviation of 0.011 Ω, corresponding to a coefficient of variation (CV) of 9.7%. Importantly, the sensor consistently returned to its baseline after each exposure, demonstrating reliable recovery behavior.

### 5.3. VOC Detection and Chromatogram Comparison

**Figure 10:**
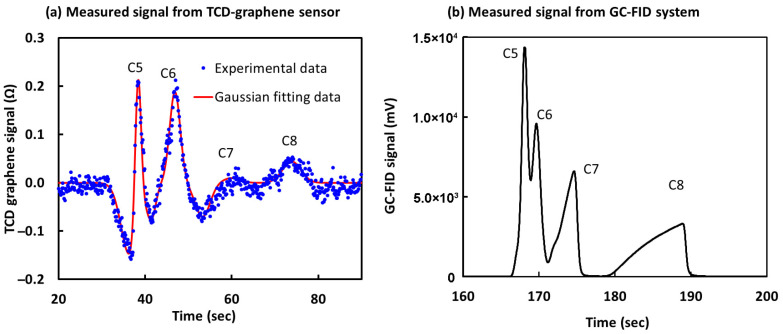
Chromatographic response of the graphene-based µTCD compared with a conventional GC-FID system. (**a**) Measured signal from the graphene µTCD showing detection of four VOCs (C5–C8), with experimental data (symbols) and Gaussian fitting (solid line). (**b**) Reference chromatogram obtained using a commercial GC-FID system under identical conditions.

Figure 10a shows the chromatogram obtained from the graphene-based µTCD, while Figure 10b presents the corresponding GC-FID reference. The µTCD successfully detects and resolves four VOCs (C5–C8) under low-power operation, with peak positions following the expected sequence governed by molecular weight and interaction strength with the stationary phase where lighter compounds traverse the column more rapidly due to weaker retention, while heavier hydrocarbons exhibit delayed arrival times. Note that the raw TCD signal exhibits baseline fluctuations and a negative pre-peak excursion, which does not reflect the true chromatographic response. This signal was therefore baseline-corrected to isolate the analyte peaks. Each peak was then fitted with a Gaussian function, and the total response was reconstructed as the sum of these components, enabling accurate extraction of peak position and amplitude while minimizing baseline-induced distortion.

The C5 peak exhibits a higher amplitude compared to heavier hydrocarbons (C6–C8). This behavior arises from a combination of transport and thermal effects. Pentane (C5), with higher diffusivity and weaker interaction with the stationary phase, propagates through the column as a narrower and more concentrated band. This results in a higher instantaneous analyte concentration at the detector, producing a stronger transient perturbation of the thermal equilibrium of the graphene bridge. Since the µTCD response is governed by heat exchange relative to the helium carrier gas, the introduction of C5 induces a larger deviation in effective heat dissipation, leading to a greater temperature rise and consequently a higher resistance response.

The chromatographic separation was further evaluated using standard parameters, including retention time and full width at half maximum (FWHM). The extracted retention times increase from approximately 38.5 s (C5) to 45.5 s (C6), 58.5 s (C7), and 70.5 s (C8), while the corresponding FWHM values increase systematically from approximately 1.6 s (C5) to 3.2 s (C6), 5.5 s (C7), and 7.5 s (C8), reflecting progressive peak broadening for heavier analytes. Heavier hydrocarbons (C6–C8) exhibited broader temporal profiles due to stronger interactions with the stationary phase, leading to increased spreading and reduced peak amplitude. The partial overlap observed between C7 and C8 is attributed to their closely spaced retention behavior. Notably, the GC-FID reference chromatogram under the same operating conditions also showed C7 and C8 resulting in close resemblance, confirming that the observed overlap originates from column-level separation rather than the intrinsic response of the µTCD. This can be validated by the fact that both GC-TCD, and GC-FID showing similar peak broadening.

### 5.4. Signal Transient and Sensitivity Analysis

**Figure 11:**
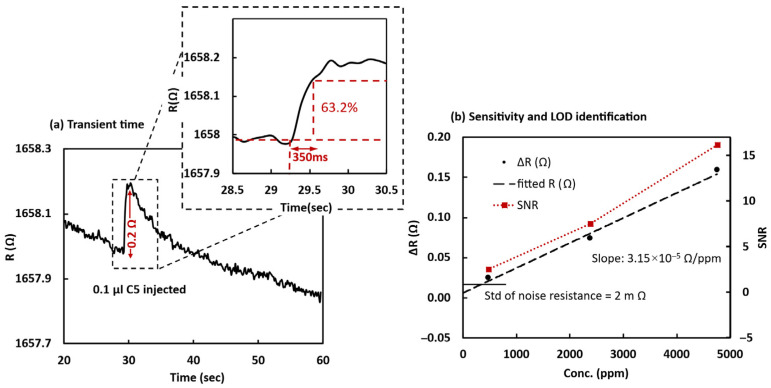
Transient and sensitivity analysis of the 20 × 825 µm^2^ graphene µTCD. (**a**) Transient response to 0.1 µL C5 injection with ~350 ms time. (**b**) ΔR and SNR versus concentration showing a sensitivity of 3.15 × 10^−5^ Ω/ppm.

Figure 11 illustrates both the transient response and sensitivity characterization of the graphene-based µTCD. As shown in Figure 10a, upon injection of 0.1 µL (~5000 ppm) pentane (C5), the device exhibits a clear ΔR of 0.2 Ω, followed by recovery toward the baseline. The inset highlights the extraction of the transient response time using the 63.2% criterion, corresponding to a first-order thermal response, yielding a response time of ~350 ms. Across multiple measurements, the transient response time was consistently observed within the range of 300–400 ms, indicating rapid thermal dynamics enabled by the low thermal capacitance of the suspended graphene structure. Because this transient is much shorter than the chromatographic peak widths measured in this work, the detector is sufficiently fast to resolve the analyte peaks without introducing significant temporal broadening.

The sensitivity and detection capability of the device are shown in Figure 10b where the ΔR exhibits an approximately linear dependence on analyte concentration over the tested range (475–4759 ppm), increasing from 0.02 Ω to 0.2 Ω with increasing concentration. This corresponded to an SNR decrease from 16.2 to 2.5 over the same range with a linear fit yielding an average sensitivity of $3.15\times 10^{-5}\;Ω/\mathrm{ppm}$. Also note that the baseline noise level was quantified by calculating the standard deviation of the pre-injection signal. To evaluate the detection limit using a conventional analytical criterion, the LOD was calculated using the 3σ/slope method,

(12) $$LOD=\frac{3ơ}{m}$$

where m is the slope of the linear fit of ΔR versus analyte concentration. Using std of baseline noise (ơ=2 mΩ) and a sensitivity of $3.15\times 10^{-5}\;Ω/ppm$, the resulting LOD is ~190 ppm. This behavior is consistent with the electrothermal sensing mechanism, where the resistance response scales with the temperature change induced by gas-dependent heat transfer.

### 5.5. Power Consumption Analysis During Chromatography

The electrical power consumption of the µTCD was determined based on Joule heating in the suspended multilayer graphene (MLG) bridge. Under DC operation, the input power is given by:

$$P=\frac{V^{2}}{R}$$

where V is the applied bias voltage across the graphene bridge and R is the measured device resistance under operating conditions. During chromatographic measurements using the $20\times 825{\;µm}^{2}$ device, a constant DC bias of 500 mV was applied across the suspended MLG bridge. The device resistance varied dynamically due to electrothermal interactions with the surrounding gas. Under steady-state operating conditions, the resistance stabilized at 1.66 kΩ, which was then used to calculate the electrical input power. Based on this measured resistance, the corresponding power consumption was ~150 µW.

### 5.6. Performance and Summary

A summary of the key performance metrics of the graphene-based µTCD is provided in Table 2 where the reported device demonstrated low-power operation. These results highlight its suitability for portable and energy-constrained gas sensing applications.

While conventional TCDs achieve higher absolute sensitivity at elevated power levels, such operating conditions are not directly applicable to suspended graphene-based devices. Previous study on suspended CVD-grown graphene has shown that applied voltages above relatively low thresholds (~0.2–0.5 V), can induce voltage-mediated delamination and breakdown of device functionality, arising from the inherent instability of fully suspended graphene under electrical bias [38]. As a result, increasing power beyond this regime can lead to structural and electrical degradation rather than improved sensing performance. Therefore, the device is intentionally operated in an ultra-low-power regime (~10^−4^ W), highlighting a distinct operating space enabled by graphene-based architectures.

In the future, such limitations could be mitigated through improved device and material design, including partial suspension of graphene instead of complete, enhanced adhesion between graphene and the supporting substrate, and the use of multilayer or hybrid graphene structures with improved thermal robustness and heat dissipation capability.

## 6. Conclusions

This paper presented the fabrication and characterization of graphene-based µTCDs utilizing freestanding MLG to detect VOCs based on the thermal property resulting in gas chromatography. The fabricated devices consisted of MLG with widths ranging from 20 µm to 40 µm and lengths from 200 µm up to 1200 µm. This was realized by the combination of (1) the modified PMMA-assisted CVD MLG transfer, and (2) the MLG patterning by channel geometry designs without photolithography.

Testing was conducted on three fabricated µTCDs with nearly identical footprints to evaluate performance across device geometries. The µTCD featuring a 30 × 550 µm^2^ free-standing MLG was first calibrated under varying temperature and carrier flow rate conditions, yielding a linear temperature coefficient of −0.16%·K^−1^ along with a positive response to increasing N_2_ flow rates up to 30 sccm. Building on this, the µTCD with a 20 × 825 µm^2^ MLG footprint demonstrated effective chromatographic detection of a C5–C8 VOC mixture within a micro gas chromatography system, producing results that closely matched those obtained from GC-FID measurements. In addition, the µTCD incorporating a 40 × 410 µm^2^ MLG footprint achieved an estimated limit of detection of 191 ppm for C5 while maintaining an average power consumption of only 151 µW. Collectively, these results demonstrate the feasibility of sub-milliwatt µTCD operation for µGC applications through the use of a large suspended MLG structure. Further improvements in stability and performance could be realized by replacing CVD graphene, which is inherently polycrystalline, with crystalline pristine graphene.

## Figures and Tables

**Table 1:** State-of-the-art gas detectors in µGCs.

	Thermal Conductivity (TCD)		Chemiresistors (CR)		Photoionization Detector (PID)		Flame Ionization Detector (FID)	
	Graphene thermistor [This work]	Metal thermistor [17,18,19,20]	Metal nanoparticles [7,8]	Functionalized graphene [9]	Discharged plasma PIDs [10]	UV-Lamp PIDs [11,12]	Plasma-assisted FID [15]	Gas-combustion [16]
Power consump.	0.15 mW	1 mW~1 W	10~500 mW	1~10 mW	1.2 W	0.5~1 W	1~5 W	1~3 W
Response time	≤1 s	≤1 s	≤1~10 s	≤1~10 s	≤1 s	≤1 s	≤1 s	≤1 s
Req. for arc generation	No	No	No	No	No	No	Yes	Yes
Size of system	<10 cm^3^	<50 cm^3^	<50 cm^3^		500 cm^3^	300 cm^3^	2000 cm^3^	1000 cm^3^
Recovery time	1.5 s	1 s	2~100 s		<1 s	<1 s	<1 s	0.5 s

**Table 2:** Specification of the graphene TCD for µGC.

Parameter	Value
Active material	Multilayer graphene (MLG)
Operating power	0.15 mW (500 mV bias)
ΔR (avg)	0.05~0.2 Ω
Transient response time	300~400 ms [in chromograms]
LOD	190 ppm (C5)
Tested linearity zone	475–4750 ppm

## Data Availability

The data presented in this study are available upon reasonable request from the primary author (farhan.sium@utah.edu).

## Supplementary Materials

The following supporting information can be downloaded at: https://www.mdpi.com/article/10.3390/s26113535/s1.
