# Supplementary material for: A Sub-Milliwatt Graphene-Based Thermal Conductivity Detector for On-Site Gas Analysis
Source: Sensors (Basel). 2026 Jun 3;26(11):3535. doi: 10.3390/s26113535 (PMC13258928; doi:10.3390/s26113535)
Supplement: Supplementary file 1 [file sensors-26-03535-s001.zip › sensors-4267624-supplementary.pdf]

Supplementary

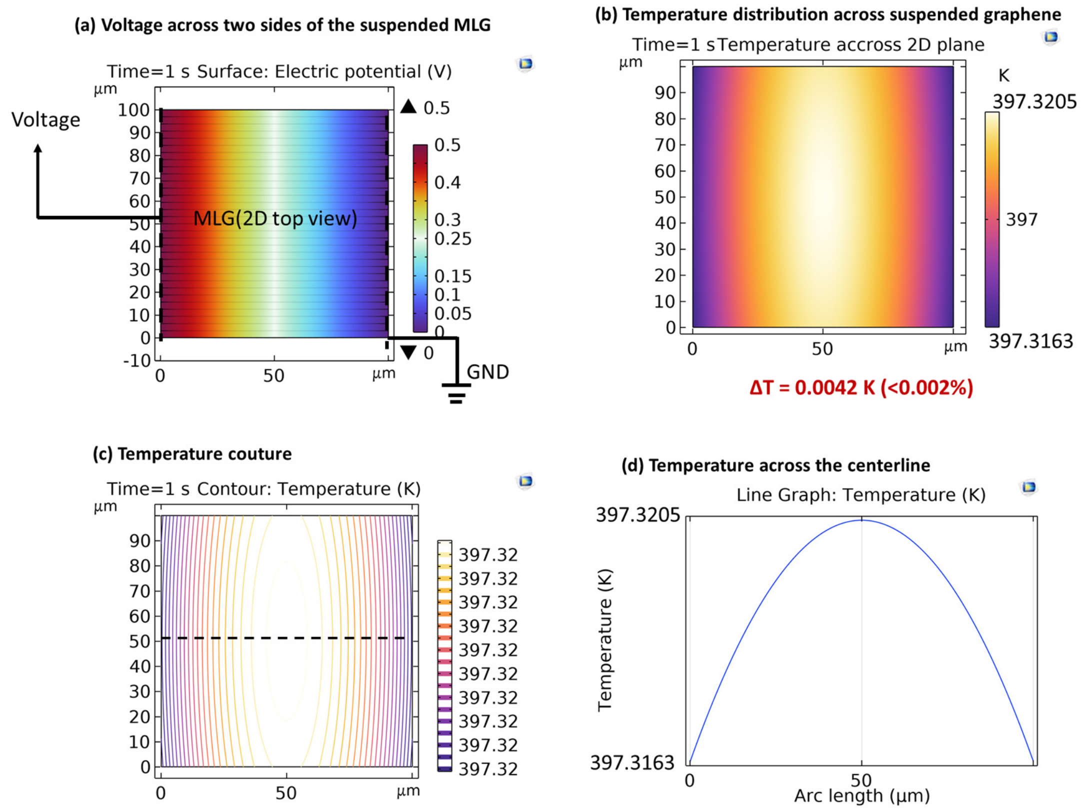

Supplementary Figure S1: Electrothermal simulation of the suspended multilayer graphene (MLG) device. (a) Electric potential distribution across the MLG, showing a uniform voltage gradient between the biased electrode and ground. (b) Temperature distribution across the 2D plane, indicating minimal spatial variation. (c) Temperature contour map with the dashed line indicating the cross-section used for line extraction. (d) Temperature profile along the indicated line, showing a smooth parabolic distribution with a total temperature variation of $\Delta T \approx 0.0042\,\mathrm{K}$ (<0.002%), confirming near-uniform thermal behavior across the graphene surface.

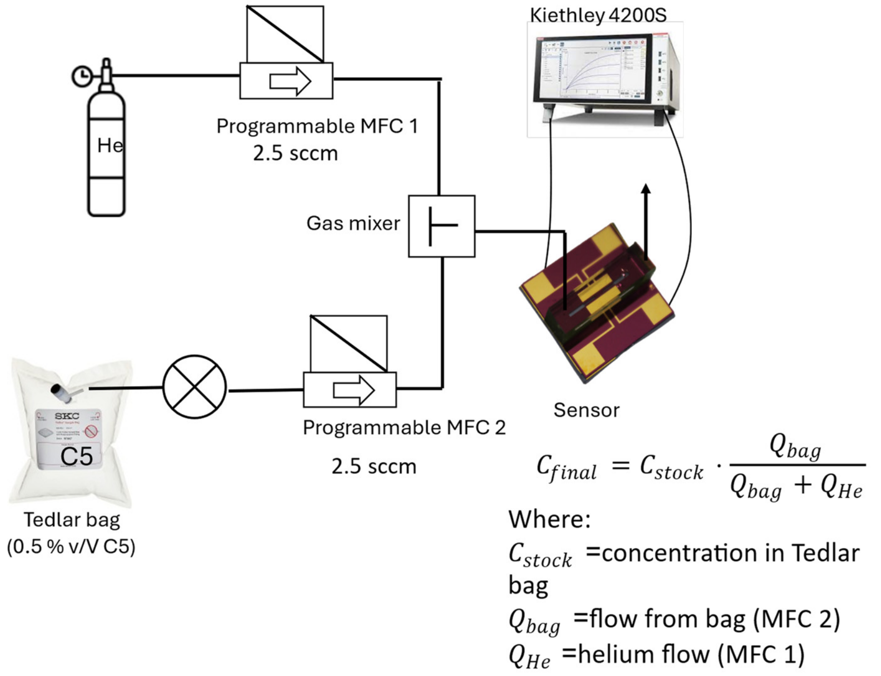

Supplementary Figure S2: Schematic of the flow-based gas sensing setup used for VOC detection in TCD used for repetitive gas test. Helium carrier gas is supplied through a programmable mass flow controller (MFC 1), while VOC vapor (C5) from a Tedlar bag (0.5% v/v) is introduced via a second programmable mass flow controller (MFC 2). The two streams are mixed in a gas mixer and delivered to the graphene-based µTCD sensor, with electrical readout performed using a Keithley 4200S system. The final gas concentration is controlled by the flow ratio and is given by $C_{final} = C_{stock} \cdot \frac{Q_{bag}}{Q_{bag}+Q_{He}}$, where C_{stock} is the concentration in the Tedlar bag, and Q_{bag} and Q_{He} are the flow rates from the bag and helium, respectively.
